# Akt-Girdin as oncotarget

**DOI:** 10.18632/oncoscience.233

**Published:** 2015-09-02

**Authors:** Masahide Takahashi, Naoya Asai, Atsushi Enomoto

**Affiliations:** Department of Pathology, and Division of Molecular Pathology, Center for Neurological Disease and Cancer, Nagoya University Graduate School of Medicine, Nagoya, Japan

**Keywords:** Akt, girdin, cancer-associated fibroblasts, tumor microenvironment

In recent years the tumor microenvironment has emerged as an important target for cancer therapy. The tumor microenvironment facilitates tumor progression and metastasis by providing a matrix for the integration of intricate networks suitable to maintain and nourish tumor cells, as well as suppress the normal immunologic antitumor defenses. However, the details of the pathways in the tumor microenvironment that provides crucial oncogenic signals and accelerates tumor growth remain unclear.

It has been well established that PI3K-Akt signaling plays a crucial role in the development, progression and metastasis of malignant tumors. We previously identified a new Akt substrate Girdin that binds the actin cytoskeleton and regulates the motility of cancer cells and fibroblasts [1, 2]. We also found that Girdin is important for postnatal angiogenesis and neurogenesis using girdin-defcient mice, regulating migration of endothelial cells and neuroblasts [3, 4].

Tumor cells recruit a diverse array of cell types to their surrounding stroma, including cancer-associated fibroblasts (CAF), endothelial cells and pericytes, that constitute tumor vessels, immune cells such as tumor-associated macrophages, and adipocytes, leading to the formation of highly complex neoplastic tissues [5]. Our immunohistochemical analysis revealed that Girdin is expressed and phosphorylated in CAF and blood vessels within the tumor microenvironment [6]. Based on these findings, we focused on roles of Akt-Girdin signaling in the tumor microenvironments. Interestingly, we found that allogeneic Lewis lung tumor tissues grown in knock-in mice defective in Akt-mediated Girdin phosphorylation (SA mice) show decreased infiltration of CAF and limited tumor growth, compared with that observed in wild-type (WT) mice. In addition, Lewis tumor tissues displayed limited growth when co-transplanted with CAF from tumor-bearing SA mice compared with CAF from tumor-bearing WT mice [6]. On the other hand, Girdin phosphorylation did not appear to affect tumor angiogenesis. Collectively, these findings revealed a role for Akt-mediated Girdin phosphorylation in CAF during tumor progression, highlighting the importance of Akt inhibition in both tumor cells and other cells that comprise the tumor microenvironments.

CAF are known to secrete various growth factors, chemokines, and cytokines and degrade extracellular matrix (ECM) proteins, thereby promoting tumor proliferation and invasion. In addition, CAF can assist in the invasion of surrounding tissue by actively remodeling the ECM, subsequently providing the routes that can be exploited by tumor cells. Thus, it is important to elucidate the mechanisms of CAF development and differentiation. Previous studies showed that mechanical forces regulated by ECM stiffness, TGF-β1 and other soluble factors secreted from tumor cells act in synergy with TGF-β1 signaling as a critical factor in CAF development [7]. However, our understanding of the mechanisms of CAF development and differentiation is far from complete. In particular, how intracellular signaling pathways regulate the dynamic cellular activities in CAF remains to be solved. Our previous studies have shown that Girdin is an actin-binding protein and its Akt-mediated phosphorylation is crucial for actin reorganization [1–3]. This notion, together with our recent finding showing high expression of Girdin in CAF in tumor tissues [6], leads to our hypothesis that Girdin integrates into thick actin cables predominantly composed of α-smooth muscle actin to regulate the contractility of CAF, which could promote ECM remodeling required for tumor growth and infiltration.

Upstream regulator(s) that induce Girdin phosphorylation in CAF are still unknown. Tumor cells secrete numerous soluble growth factors, cytokines, and chemokines, including TGF-β1, hepatocyte growth factor, and SDF-1 whose cognate receptors are expressed on CAF [8]. In fact, our study suggested TGF-β1 and PDGF as candidate growth factors that govern Girdin function because fibroblast and CAF isolated from SA mice were defective in TGF-β1 and PDGF-induced migration [6]. A signaling network regulated by synergy or crosstalk with tumor cells should be extensively studied in the future work.

The components of the tumor stoma including CAF are heterogeneous depending on individual tumors, and this tumor tissue diversity makes it difficult to treat cancer patients uniformly. In addition, a variety of microenvironmental factors are involved in the tumor progression, and synergistic or additive effects of Girdin phosphorylation with those factors could be important for clinical outcome of the patients. Our findings suggest a novel mechanism whereby Akt signaling is central to both tumor cells and cells that constitute the tumor microenvironment. This study provides a basis for the potential targeting of Akt-Girdin signaling within the tumor microenvironment to develop novel therapies against human cancer.
